# A comparison of the recording of comorbidity in primary and secondary care by using the Charlson Index to predict short-term and long-term survival in a routine linked data cohort

**DOI:** 10.1136/bmjopen-2015-007974

**Published:** 2015-06-05

**Authors:** C J Crooks, J West, T R Card

**Affiliations:** Division of Epidemiology and Public Health, Nottingham City Hospital, University of Nottingham, Nottingham, Nottinghamshire, UK

**Keywords:** EPIDEMIOLOGY, GASTROENTEROLOGY, GENERAL MEDICINE (see Internal Medicine), PRIMARY CARE, STATISTICS & RESEARCH METHODS

## Abstract

**Objective:**

Hospital admission records provide snapshots of clinical histories for a subset of the population admitted to hospital. In contrast, primary care records provide continuous clinical histories for complete populations, but might lack detail about inpatient stays. Therefore, combining primary and secondary care records should improve the ability of comorbidity scores to predict survival in population-based studies, and provide better adjustment for case-mix differences when assessing mortality outcomes.

**Design:**

Cohort study.

**Setting:**

English primary and secondary care 1 January 2005 to 1 January 2010.

**Participants:**

All patients 20 years and older registered to a primary care practice contributing to the linked Clinical Practice Research Datalink from England.

**Outcome:**

The performance of the Charlson index with mortality was compared when derived from either primary or secondary care data or both. This was assessed in relation to short-term and long-term survival, age, consultation rate, and specific acute and chronic diseases.

**Results:**

657 264 people were followed up from 1 January 2005. Although primary care recorded more comorbidity than secondary care, the resulting C statistics for the Charlson index remained similar: 0.86 and 0.87, respectively. Higher consultation rates and restricted age bands reduced the performance of the Charlson index, but the index's excellent performance persisted over longer follow-up; the C statistic was 0.87 over 1 year, and 0.85 over all 5 years of follow-up. The Charlson index derived from secondary care comorbidity had a greater effect than primary care comorbidity in reducing the association of upper gastrointestinal bleeding with mortality. However, they had a similar effect in reducing the association of diabetes with mortality.

**Conclusions:**

These findings support the use of the Charlson index from linked data and show that secondary care comorbidity coding performed at least as well as that derived from primary care in predicting survival.

**Strengths and limitations of this study**

- Large population-based study using linked primary care, secondary care and national death register data to provide complete longitudinal health records.
- Detailed stratified assessment of the performance of the Charlson index with 5 years of follow-up, including the effect of the consultation rate.
- Comparison of primary and secondary care comorbidity data in adjusting for case mix in survival analyses, and the benefits of linking the two.
- Large routine data sets might have misclassification errors in coding. While data cleaning and audits confirm overall accuracy, it is not possible to directly validate Hospital Episodes Statistics coding due to its anonymisation prior to research use.

## Introduction

Comparisons of outcomes from hospitals frequently use the comorbidity data recorded during a hospital admission to adjust for differences in case mix.^1–4^ However, secondary care admissions provide only snapshots of a patient's clinical journey and might not contain comprehensive information on chronic conditions. In contrast, primary care data records a longitudinal picture of a patient's clinical journey, but some events managed directly in secondary care might be missed. Previous work provides evidence to support this hypothesis by showing that primary care data increased the sensitivity of detecting diagnoses (eg, diabetes, cirrhosis, acute coronary syndrome and upper gastrointestinal bleeding), while secondary care data identified cases with a higher mortality.^5–8^ Combining primary and secondary data care might therefore provide a better adjustment for differences in case mix than data from single clinical settings.

One common example of a secondary care-derived comorbidity score used to adjust for case-mix differences is the Charlson index. It was originally derived in 1987 using a secondary care cohort of just 559 medical admissions with 1 year of follow-up for death, and it was validated in only 685 patients with breast cancer.^9^ Yet, despite the small number of patients and the passage of time, the Charlson index remains the most widely validated and used comorbidity score in research.^10^ What is not clear, however, is whether the Charlson index performs equally well in primary and secondary care data over both the short (1 year) and longer term (up to 5 years), and whether combining information from both sources would provide a better measure of comorbidity. With the recent linkage between primary and secondary care records in England, and the additional linkage to the Office for National Statistics death register, there is now an opportunity to assess the performance of the Charlson index in these different clinical settings while using the same underlying population cohort.

In this study, we aim to assess the performance of the Charlson index derived from either primary or secondary care data or both. We will assess how changing the parameters that were used to define the comorbidities for the Charlson index affects its performance, and how the Charlson index interacts with patients’ age, socioeconomic status and consultation rates. The performance will be assessed with regard to discrimination in predicting short-term and long-term mortality and adjusting for case mix. For the latter, we will assess the association of both a chronic diagnosis of diabetes and an acute secondary care diagnosis of acute upper gastrointestinal bleeding with mortality, by adjusting for comorbidity defined by the Charlson index derived from its coding in either primary or secondary care.

## Methods

### Data source

A cohort study was designed using linked longitudinal data from the linked English Hospital Episodes Statistics (HES) data, Clinical Practice Research Datalink (CPRD) and Office of National Statistics (ONS) death register. These data have linked records of all primary care events, hospital admissions and causes of death from 1 April 1997 to 31 December 2010 for 3% of the English population. Owing to the comprehensive English primary care system, the population registered to each practice contributing to the CPRD is broadly representative of the general English population, but there is some under-representation of teenagers and young adults who are generally more mobile and registered as temporary patients.^11^ The data sources are subject to quality checks, and a practice's data are only used when they are of high enough quality to be used in research. This is referred to as the up to research standard time period and is defined separately for each primary care practice.

### Study population

We selected all adults older than 20 years in the CPRD on 1 January 2005 who were registered at a practice that continued contributing to the CPRD until 1 January 2010, and had consented to linkage with HES and ONS. These patients were followed up until the earliest of date of death, the date a patient's care was transferred out of a CPRD practice or the last date of the study (1 January 2006 or 1 January 2010, depending on the analysis).

### Exposures

The main exposure of interest for the study was the Charlson index (table 1). This was calculated three times for each person using: (1) Read codes recorded in primary care, (2) International Classification of Diseases (ICD) 10 codes recorded in secondary care and (3) by combining both the Read and ICD10 codes. Diagnostic codes were only included prior to 1 November 2004 in order to exclude codes that might have been directly related to terminal events in the initial follow-up period. The original derivation of the Charlson index was scaled so that a single point increase in the score was equivalent to a reduction in survival by a decade.^9^ We therefore grouped all scores greater than 5 (as in the original paper), because differentiating between reductions in survival greater than 50 years was not judged to be useful. We used the ICD10 codes previously published by Quan *et al*^12^ and the Read codes were adapted from the Read and Oxmis code lists of Khan *et al.*^13^

**Table 1:** Comorbidities contributing to the original Charlson index

Comorbidity	Weighting
Myocardial infarct	1
Congestive heart failure	1
Peripheral vascular disease	1
Cerebrovascular disease	1
Dementia	1
Connective tissue disease	1
Chronic pulmonary disease	1
Ulcer disease	1
Mild liver disease	1
Diabetes	1
Hemiplegia	2
Moderate or severe renal disease	2
Diabetes with end-organ damage	2
Any tumour	2
Leukaemia	2
Lymphoma	2
Moderate or severe liver disease	3
Metastatic solid tumour	6
HIV	6

Other exposures of interest included age (in 10-year age bands), gender, primary care consultation rate (categorised into 0, 1, 2–3, 4–7, 8–13, ≥14 primary care face-to-face consultations a year), socioeconomic quintiles defined by Indices of Multiple Deprivation for England 2007,^14^ and hospitalisation in the previous year defined by a recorded admission in secondary care data (excluding the 60 days prior to the study start date).

### Outcome

The outcome of the study was all-cause mortality. The date and fact of death were obtained from the linked Office for National Statistics data.

### Analysis

The proportion of the study population with a Charlson index greater than zero was calculated. Cox proportional hazard models predicting survival up to 1 year were then fitted for each derivation of the Charlson index. Age and gender were included as a priori confounders in the models. The interaction of the Charlson index was tested (using likelihood ratio tests) with age, socioeconomic status and consultation rate. The follow-up time was then extended to 5 years, and the model stratified by follow-up time (<1, 1–2, 2–3, 3–4, 4–5 years).

To compare goodness-of-fit between the different models, we used Akaike's Information Criterion which penalises the likelihood for model complexity. Likelihood ratio testing cannot be used, as models with the different Charlson index derivations are not nested within each other. To assess the discriminative ability of each of the Charlson indexes in relation to survival, we derived Harrell's C statistic.^15^ This was calculated for each derivation of the Charlson index, and then stratified for the different follow-up times, ages, socioeconomic quintiles and consultation rates described above. CIs for C statistics and differences were calculated from jackknife variance estimates.^16^

We performed three sensitivity analyses. First, to assess the effect of only using recently recorded comorbidity, we derived the Charlson index from either comorbidity recorded only in the year prior to the study, or comorbidity recorded more than 1 year prior to the study. Second, to assess the effect of potentially including codes related to palliative end-of-life processes, we excluded patients with less than 1, 3, or 6 months of follow-up after the study start date. Finally, it has been shown that recording of prevalent diagnoses can take up to 1 year following registration with a primary care doctor.^17^ We therefore repeated the analysis excluding patients with less than 1 year of data prior to the start date of the study.

### Performance of the Charlson index in adjusting for comorbidity in diabetes and upper gastrointestinal bleeding

The extent to which the Charlson index reduces the direct effect of an association with mortality is a measure of the extent it adjusts for the indirect effect of comorbidity. We selected two diseases as examples for which this is plausible; diabetes, which is a disease that increases the risk of many other comorbidities that indirectly reduce survival,^18^ ^19^ and upper gastrointestinal bleeding, which is an acute event in which comorbidity both predicts its occurrence and its subsequent mortality.^20–22^ We used a Cox proportional hazards model adjusted for age and gender to compare the ability of each of the Charlson index derivations to adjust for the effect of comorbidity on 1-year survival. For the analysis of diabetes, we removed the category of diabetes from the calculation of the Charlson index to avoid duplicating its coefficient in the model.

For a chronic disease such as diabetes, the diagnosis date does not necessarily indicate disease onset; therefore, for this analysis, we selected all patients with a recording of diabetes prior to November 2004, and followed them up from 1 January 2005 (using the same definition as when defining the Charlson index). This cohort was compared with all patients without a recording of diabetes prior to November 2004 and followed up from 1 January 2005.

In contrast, upper gastrointestinal bleeding is an acute event with a defined date of onset. We therefore followed up all patients with a first recorded bleed in our cohort from the date of bleed using a method we published previously.^6^ For the comparison cohort of patients without upper gastrointestinal bleeding, we followed up from a random observed date. The Charlson index was recalculated on either the date of bleed or a random observed date for the bleed and non-bleed patients, respectively.

All analyses were performed using Stata V.13.

## Results

### Study population

A total of 657 264 people were followed up from 1 January 2005 until 1 January 2006, or 1 January 2010, depending on the analysis. In the first year of follow-up, there were 21 672 deaths during 216 million days, with an average follow-up of 329 days/person. For the extended follow-up to 2010, there were 95 596 deaths in 1.9 million years with an average follow-up of 2.9 years/person. The age structure of the population was similar to that of the UK population (table 2), as would be expected from a national population database.^11^

**Table 2:** Demographics of the study population, England, 2005–2010

	Number of people	Percentage of total
Age group (years)		
20–29	136 464	21.34
30–39	136 464	20.76
40–49	92 744	14.11
50–59	74 659	11.36
60–69	61 373	9.34
70–79	64 470	9.81
80–89	67 004	10.19
≥90	20 267	3.08
Gender		
Male	328 382	49.96
Female	328 882	50.04

Charlson index score	Derived from Read codes (n)	(%)	Derived from ICD10 codes (n)	(%)	Derived from combined codes (n)	(%)
0	467 501	71.13	573 047	87.19	456 681	69.48
1	111 178	16.92	42 660	6.49	110 692	16.84
2	42 246	6.43	21 816	3.32	42 579	6.48
3	19 680	2.99	8619	1.31	21 641	3.29
4	8406	1.28	4109	0.63	10 574	1.61
≥5	8253	1.26	7013	1.07	15 097	2.30

ICD, International Classification of Diseases.

### Recording of the Charlson index comorbidity in primary and secondary care

Table 2 shows the proportion of the population with a Charlson index greater than zero when derived from either a single data set or from both. Read codes from primary care identified almost three times the number of people with a Charlson index greater than zero compared to when the Charlson index was derived from hospital admission ICD codes. When examined in more detail, the primary care data identified more of each Charlson index comorbidity than did secondary care data, apart from metastatic cancer and hemiplegia (see online supplementary table A). Thirty per cent of people had a Charlson index greater than zero when the information from both data sets was combined.

### Fit and performance of the Charlson index in predicting mortality

Age contributed the most to an improvement in both goodness-of-fit and discrimination of the Cox proportional hazards model (table 3). There was a better goodness-of-fit when the Charlson index was derived from secondary care ICD10 codes than when it was derived from primary care Read codes. However, the slight improvement of using ICD10 codes over Read codes was reduced when an indicator variable was included for a recent hospitalisation in the Read code only version.

**Table 3:** Performance and goodness-of-fit of different proportional hazard models predicting mortality using different demographics and derivations of the Charlson index, England, 2005–2010

Model covariates	C statistic	95% CI	Difference in C statistic from previous row*	AIC
Gender only	0.513	(0.509 to 0.516)	–	576 065
Age and gender	0.844	(0.842 to 0.846)	0.332 (0.328 to 0.335)	538 291
Age, gender, and Read-derived Charlson index	0.861	(0.859 to 0.863)	0.016 (0.016 to 0.017)	534 185
Age, gender, recent hospitalisation and Read-derived Charlson index	0.868	(0.866 to 0.869)	0.0069 (0.0063 to 0.0074)	532 176
Age, gender and ICD10-derived Charlson index	0.870	(0.868 to 0.872)	0.0026 (0.0018 to 0.0034)	531 085
Age, gender, recent hospitalisation and ICD10-derived Charlson index	0.872	(0.871 to 0.874)	0.0021 (0.0018 to 0.0024)	530 318
Age, gender and combined ICD10 and Read-derived Charlson index	0.869	(0.869 to 0.870)	−0.0039 (−0.0046 to −0.0031)	531 830
Age, gender, recent hospitalisation and combined ICD10 and Read-derived Charlson index	0.873	(0.871 to 0.874)	0.0040 (0.0036 to 0.0044)	530 469

The smaller the AIC value, the better the goodness-of-fit with less information loss.

*All differences in C statistic in this column had a p value <0.0001.

AIC, Akaike's Information Criterion; ICD, International Classification of Diseases.

There was no large difference in the discrimination of the model for overall survival, whichever codes were used to derive the Charlson index: Read codes from primary care (Harrell's C statistic 0.86), ICD10 codes from secondary care (C statistic 0.87), or both Read and ICD10 codes (C statistic 0.87). Including a marker for a recent hospital admission resulted in a slightly improved discrimination for each Charlson derivation (p<0.0001).

### Stratified analysis of the fit and performance of the Charlson index in predicting mortality

There was a significant interaction between age group and Charlson index (likelihood ratio test p<0.0001) with the model's discrimination reducing with age. Each Charlson index derivation had a good discrimination for each of the 10-year age bands younger than 70 years, fair discrimination for each of the 10-year age bands up to 90 years, but was poor at discriminating for those who were older (table 4). The Charlson index derived from ICD10 or both data sources showed small but significant improvements (p<0.0001) in discrimination within the age bands older than 50 years, when compared with the primary care-derived Charlson index.

**Table 4:** The discrimination of Charlson index in predicting mortality stratified by age, follow-up and consultation rate, England, 2005–2010

Age (years)	Number of people	(%)	C statistic adjusted for recent hospitalisation, gender and Charlson index derived from		
			Read (95% CIs)	ICD10	ICD10 and Read
20–29	140 283	21	0.71 (0.66 to 0.75)	0.71 (0.67 to 0.76)	0.71 (0.67 to 0.76)
30–39	136 464	21	0.72 (0.69 to 0.76)	0.72 (0.69 to 0.75)	0.74 (0.70 to 0.77)
40–49	92 744	14	0.73 (0.71 to 0.75)	0.72 (0.70 to 0.75)	0.74 (0.72 to 0.76)
50–59	74 659	11	0.75 (0.74 to 0.76)	0.75 (0.73 to 0.76)	0.77* (0.75 to 0.78)
60–69	61 373	9	0.72 (0.71 to 0.73)	0.71 (0.69 to 0.72)	0.73* (0.72 to 0.74)
70–79	64 470	10	0.66 (0.65 to 0.66)	0.67* (0.66 to 0.67)	0.67 (0.66 to 0.68)
80–89	67 004	10	0.62 (0.61 to 0.62)	0.63* (0.63 to 0.64)	0.63 (0.63 to 0.64)
≥90	20 267	3	0.57 (0.56 to 0.58)	0.58* (0.57 to 0.59)	0.58 (0.57 to 0.59)

Consultations per year (n)	Number of people	(%)	C statistic adjusted for age, recent hospitalisation, gender and Charlson index derived from		
			Read	ICD10	ICD10 and Read
0	139 457	21	0.87 (0.86 to 0.88)	0.88 (0.86 to 0.89)	0.87 (0.86 to 0.89)
1	70 216	11	0.90 (0.89 to 0.91)	0.90 (0.89 to 0.91)	0.90 0.89 to 0.91)
2–3	105 686	16	0.90 (0.89 to 0.90)	0.90 (0.89 to 0.91)	0.90 (0.89 to 0.91)
4–7	134 415	20	0.86 (0.86 to 0.87)	0.87* (0.86 to 0.87)	0.87 (0.86 to 0.87)
8–13	102 553	16	0.80 (0.80 to 0.81)	0.81* (0.81 to 0.82)	0.81 (0.81 to 0.82)
≥14	104 937	16	0.72 (0.71 to 0.72)	0.74* (0.73 to 0.74)	0.73 (0.73 to 0.74)

Year of follow-up	Number of people	Follow-up (years)	C statistic adjusted for age, recent hospitalisation, gender and Charlson index derived from:		
			Read	ICD10	ICD10 and Read
0–1	657 264	590 375	0.87 (0.87 to 0.87)	0.87* (0.87 to 0.87)	0.87 (0.87 to 0.87)
1–2	527 516	469 662	0.85 (0.85 to 0.85)	0.85* (0.85 to 0.85)	0.85* (0.85 to 0.85)
2–3	413 085	360 469	0.83 (0.83 to 0.84)	0.84 (0.83 to 0.84)	0.84* (0.83 to 0.84)
3–4	311 556	266 840	0.82 (0.82 to 0.83)	0.82 (0.82 to 0.83)	0.83* (0.82 to 0.83)
4–5	226 541	190 162	0.81 (0.81 to 0.81)	0.81 (0.81 to 0.81)	0.81* (0.81 to 0.81)

*p<0.0001 for difference in C statistic from previous column.

ICD, International Classification of Diseases.

There was also a significant interaction with the consultation rate (likelihood ratio test p<0.0001) with excellent discrimination of each Charlson index derivation when the number of consultations in the prior year was below 14, and good discrimination above 14 consultations in the prior year (table 4). The Charlson index derived from ICD10 or both data sources showed small but significant improvements in discrimination (p<0.0001) within the strata with more than four consultations per year when compared with the primary care-derived Charlson index. There was a weak interaction with socioeconomic status (p=0.01), but when stratified, the variation was minimal with no clear trends (see online supplementary table B).

Finally, we assessed the performance of each Charlson index derivation during each year of follow-up. The C statistic remained excellent for both primary and secondary care up to 5 years of follow-up, with a slight reduction from 0.87 in the first year to 0.82 in the fifth year (table 4 combined primary and secondary care). There were very small but still significant improvements in discrimination (p<0.0001) when using either the Charlson index derived from ICD10 or both data sources. Over all 5 years of follow-up, the combined Charlson index had a C statistic of 0.85.

### Sensitivity analyses

Restricting comorbidity to that recorded only in the year prior to the study (2004) reduced the overall C statistic for the combined index to 0.84, and excluding comorbidity recorded in that year also reduced it to 0.84. Excluding patients with limited follow-up had a minimal effect on discrimination of the combined index (C statistic 0.87 for excluding 1 month and 0.86 for excluding 3 or 6 months). Finally, excluding patients who had less than 1 year of follow-up from their registration date prior to the start of the study did not alter the discrimination of the model (C statistic 0.87).

### Performance of the Charlson index in adjusting for comorbidity in diabetes and upper gastrointestinal bleeding

The effect of an upper gastrointestinal bleeding event or a chronic diagnosis of diabetes on all-cause mortality is shown in table 5 adjusted for the different derivations of the Charlson index. The derivation from ICD10 or Read codes had the same effect in reducing the direct association of diabetes with mortality. Combining the ICD10 and Read codes did have a greater effect, but the CIs overlapped with estimates for the separate derivations.

**Table 5:** HRs for the effect of a first upper gastrointestinal bleed or a diagnosis of diabetes on 1 year mortality, England, 2005–2010

Model covariates	Adjusted HR	95% CI
Upper gastrointestinal bleeding		
Age and gender only	5.34	(5.19 to 5.57)
Age, gender and Read-derived Charlson index	4.45	(4.30 to 4.61)
Age, gender, recent hospitalisation and Read-derived Charlson index	4.18	(4.03 to 4.32)
Age, gender and ICD10-derived Charlson index	3.99	(3.85 to 4.13)
Age, gender, recent hospitalisation and ICD10-derived Charlson index	3.90	(3.76 to 4.03)
Age, gender and Combined ICD10 and Read-derived Charlson index	4.11	(3.97 to 4.25)
Age, gender, recent hospitalisation and combined ICD10 and Read-derived Charlson index	3.94	(3.80 to 4.08)
Diabetes		
Age and gender only	1.34	(1.29 to 1.40)
Age, gender and Read-derived Charlson index	1.18	(1.14 to 1.23)
Age, gender, recent hospitalisation and Read-derived Charlson index	1.14	(1.10 to 1.18)
Age, gender and ICD10-derived Charlson index	1.15	(1.11 to 1.19)
Age, gender and combined ICD10 and Read-derived Charlson index	1.13	(1.09 to 1.18)
Age, gender, recent hospitalisation and combined ICD10 and Read-derived Charlson index	1.10	(1.06 to 1.15)

ICD, International Classification of Diseases.

In contrast, for upper gastrointestinal bleeding, the ICD10 codes on their own had a greater effect than Read codes in reducing the direct association with mortality. However, when a flag for a recent hospitalisation was included, the effect of the Read code-derived Charlson index became comparable with that derived from ICD10 codes. A flag for a recent hospitalisation was significantly associated with an improvement in model fit with both bleeding and diabetes independently of the Charlson index derived from either ICD10 and Read codes (likelihood ratio test for both: p<0.0001).

## Discussion

The primary and secondary care-derived Charlson comorbidity index had good discrimination for both short-term and long-term survival, with a C statistic of 0.87 in the first year and 0.85 for all 5 years. However, while comorbidity recorded in primary care identified almost three times the burden of comorbidity compared to the diagnoses recorded in secondary care, this did not produce the expected improvements in discrimination for overall survival modelling or case mix adjustment. Diagnoses recorded in secondary care actually showed a slight improvement in goodness-of-fit compared to that in primary care alone, but this was mostly comparable with the inclusion of a flag for a recent hospitalisation regardless of the reason for admission. This indicates that life-limiting comorbidity is more likely to result in a hospital admission and therefore be coded in secondary care, compared to comorbidity recorded solely in primary care.

We found larger differences in the performance of the Charlson index derivations when adjusting for comorbidity-associated mortality in specific disease situations. Upper gastrointestinal bleeding is diagnosed and managed in secondary care,^23^ and we found that comorbidity coding in secondary care or a flag for hospital admission had a greater effect in adjusting for comorbidity-associated mortality than primary care Read codes alone. In contrast, diabetes is a chronic disease that may be diagnosed and managed in primary care, and we found that comorbidity coding in primary care had the same effect on adjusting for comorbidity-associated mortality as secondary care comorbidity coding.

The strength of this study is its size and reflection of real-life practice and coding. This has allowed us to examine the performance of the Charlson index in relation to mortality in far more detail than has previously been possible, providing much needed information on its practical use, particularly regarding the timing of included codes to which we found the Charlson index was reassuringly robust. In addition, our study has examined the Charlson index separately for each year over an extended period of follow-up and therefore provided useful evidence supporting the validity of using the index for longer time periods.

Our study does share the same potential weaknesses of any study using routine data. While its large size reduces the risk of random error, there might be another potential bias in the recording of routine data through the selection of the population. However, by using a population-based database from the defined patient population registered to CPRD practices, any selection bias would be limited to issues with access to primary care. As England has universal coverage of primary care registration, this bias should therefore be minimal. By contrast, any bias related to diagnosis miscoding is more critical, and one of the limitations of routine data is that their anonymisation prevents the large-scale validation of individual records against the original clinical chart records. Although this potentially leaves the databases susceptible to accusations of poor coding quality,^24^ ^25^ the HES data submissions are regularly cleaned and monitored for data quality and consistency. An in-depth government audit of samples of UK hospital data confirmed that there was an accuracy in recording that approached 90%.^26^ Similarly, CPRD primary care records undergo regular quality and consistency checks, and a practice's data is only included when it is of high enough quality to be used in research (at these times, the data are said to be ‘up to standard’).^27^ The CPRD has been extensively validated with paper records for a wide range of diagnoses with a mean positive predictive value of 89%.^28^ The use of these routine data in research is therefore unsurprisingly considered valid in the numerous studies that utilise them. Furthermore, there is no other practical method to achieve the same scale, generalisability and efficiency in research that using routine healthcare records provides.

Previous studies have shown that using linked primary and secondary care data improved the identification of a limited number of diseases compared to using unlinked data sources. Specifically, it increased the sensitivity of identifying chronic diseases such as diabetes,^8^ ^29^ cirrhosis,^7^ venous thromboembolic events,^30^ acute myocardial infarction^5^ and acute upper gastrointestinal bleeding.^6^ In contrast, using only primary care data reduced the severity of the diagnoses identified, and missed identifying more severe cases found only in hospital data.^6^ ^7^ ^31^ Our study has now been able to generalise these previous findings by showing that primary care data do identify a greater proportion of patients with comorbidity, but that they had a lower burden of comorbidity than patients identified in secondary care data. The inclusion of this additional comorbidity from primary care did not therefore have a large effect on model performance.

A few other studies, including the one by the original author, have validated the Charlson index in discriminating survival for longer time periods.^9^ ^13^ ^32^ ^33^ However, these studies have important limitations; such as only using primary or secondary care data, restricting to patients with cancer or the elderly,^9^ ^13^ ^32^ or using methods that do not take account of censoring in routine data. Most other studies have only used inpatient mortality or 1-year mortality.^34^ In contrast, our study has been able to examine in detail the performance of the Charlson index in discriminating survival over 5 years of follow-up.

Two papers from Medicare data found no difference in discrimination from using inpatient or outpatient claims data to derive the Charlson index for predicting mortality. Zhang *et al*^35^ found that the choice of source data or time span used did not alter the discrimination for 2-year survival (C statistic=0.70). Li *et al*^36^ found that excluding outpatient claims data from the Charlson index did not alter discrimination for 6-month survival (C statistic=0.74). This contrasts with our study, but reflects the effect of restricting to an older population who had all been recently hospitalised.

A patient's presentation to healthcare of one condition increases the likelihood that other diagnoses will be identified. Therefore, one potential problem of comorbidity scores in routine data is that a comorbidity score might be a marker of patients who visit their doctors frequently.^33^ In this study, we have demonstrated that the Charlson index predicted mortality independently of consultation rates, and with excellent discrimination, albeit with a reduction to good among patients who visited their doctor more than 14 times in a year. However, this was because a greater proportion of patients with higher consultation rates had serious or multimorbidity, and this reduced the ability of the Charlson index to discriminate between the patients. A previous study found that increasing the observation period from which the Charlson index was calculated increased the sensitivity of the detection of comorbidity, but that the specificity remained the same.^37^ In our study, restricting to or excluding the year prior to the study in the calculation of the Charlson index reduced the discrimination as measured by the C statistic.

In all our models, age remained the biggest predictor of mortality. This is because age remains an accurate, comprehensive and objectively recorded variable in routine data that is strongly associated with the risk of death. In contrast, comorbidity scores in routine data attempt to simplify what will always be a complicated and imprecise clinical assessment in a restrictive coding structure.^10^ ^31^ However, in our study, the Charlson index still provided significant additional improvements in model discrimination, and also measurable improvements in performance when adjusting mortality for comorbidity as shown in the disease-specific examples of upper gastrointestinal bleeding and diabetes.

In conclusion, our study demonstrates good performance of the Charlson index using linked primary and secondary care data to classify risk of death at 1 year and for longer periods up to 5 years. We found that primary care data identified a higher prevalence of comorbidity than secondary care, but this did not result in improved performance or discrimination. The inclusion of a marker for recent hospitalisation made the data sources more comparable, but combining the data sources did not result in the expected improvement in the ability of the Charlson index to predict survival and adjust for case mix.
